# A Big Data-Driven Approach to Analyze the Influencing Factors of Enterprise's Technological Innovation

**DOI:** 10.1155/2022/3785685

**Published:** 2022-01-25

**Authors:** Qianqian Zhang

**Affiliations:** School of Information, Beijing Wuzi University, Beijing 101149, China

## Abstract

A data-driven intelligent analysis method is proposed in this paper to explore and identify the enterprise's technological innovation influencing factors. Questionnaire surveys or expert interviews are usually adopted by the traditional evaluation methods for indicators of technological innovation selection. However, it inevitably involves human factors and experts' subjective judgments, which may affect the result of enterprises evaluation. The research presents an improved text clustering method based on a semantic concept model to explore and analyze the key influencing factors of enterprise's technological innovation. The study collects textual data from 400 enterprises in Beijing and smart analyzes the critical influencing factors of enterprise's technological innovation by using the proposed method. The influencing factors can be divided into seven categories. In addition, compared with the traditional K-means clustering method, the proposed method has a good effect. We proposed a methodology to conduct an intelligent analysis for enterprise's technological innovation under the data-driven. It can provide more objective and auxiliary suggestions for the evaluation of the enterprise's technology innovation.

## 1. Introduction

Technological innovation is the foundation of the survival and development of enterprises and the driving force for the country's economic and social development. It is essential for an enterprise to gain a competitive advantage by correctly analyzing and evaluating the technological innovation capability. Since the middle of the 18th century, the world has experienced three industrial revolutions. Social development is entering the era of data revolution with the development and application of new-generation information technologies such as cloud computing, big data, mobile Internet, artificial intelligence, and the Internet of Things. The production and circulation of massive data gave birth to “big data” and set off the fourth industrial revolution.

The digital economy era has given birth to new production factors represented by big data. Data-driven continuous growth and innovative development are the main lines of enterprise digital transformation. Compared with the past process-driven, data-driven allows companies to use massive and multidimensional data to establish a more comprehensive evaluation system, create direct business innovation growth, and continuously improve operational efficiency. It is an essential means to maintain sustainable development in market competition. With the continuous development of enterprises, various forms of technical documents and text information continue to spew. According to statistics, 80% of enterprise data exist in unstructured conditions, such as Web pages, technical papers, e-mails, etc. Especially in enterprise's technological innovation, related technological innovation activity reports, meeting minutes, annual corporate reports, patent technology files, project application reports, and textual information are increasing with each passing day. Most of the time, enterprises often need to deal with disordered unstructured textual data except structured data. Ignoring the text information generated by corporate technological innovation activities will inevitably affect the result of technical innovation management. Enterprises need to deal with these collected files and explore the value and knowledge behind these massive amounts of data. Therefore, the rise of big data and the development of intelligent text analysis technology have used many unstructured and fragmented textual data that enterprises initially neglected. However, it is a significant challenge for the era of big data to mine knowledge from massive unstructured data and provide auxiliary decision-making for enterprise technological innovation.

Text mining generally refers to the process of extracting valuable, nontrivial patterns or knowledge from a large amount of unstructured or semi-structured text files. Therefore, text mining provides an effective measure for textual data collation, analysis and, mining. It meets the massive demand for processing and analyzing an enormous amount of unstructured and semi-structured textual data. To a certain extent, it solves the labour and material cost problems of manual text processing. Text mining, as a representative of the intelligent measure method, has been widely applied in various fields. For example, the evaluation of business intelligence and enterprise technology analysis [1–3], the enterprise technology opportunity identification [4, 5], correlation analysis of enterprise technology cooperation behavior [6–8], the analysis of enterprises technology maturity [9–11], and prediction of enterprise technology development trend, etc. [12–14].

The intelligent text mining algorithm can quickly and high-quality organize amounts of information into a few meaningful clusters and obtain the hidden potential knowledge or patterns. The rapid growth of textual data, especially in enterprise's technological innovation, has become diverse, high-dimensional, complex data loaded with semantic information. Therefore, it is feasible to explore the essential influencing factors of enterprise's technological innovation based on intelligent analysis algorithms and realize the semantic organization for technological innovation evaluation. This paper collects technical data from 400 enterprises in Beijing. It combines the proposed intelligent text clustering algorithm to realize knowledge mining and acquire enterprise's technological innovation at the semantic level.

The remainder of this paper is organized as follows. The second section presents the related works. The third section provides the implementation process of the proposed methodology and makes a performance comparison validation with the traditional K-means clustering method. While section four presents the result and the section five give a discussion of this research. In the final section, we conclude this paper's work.

## 2. Related Works

The previous studies of enterprise's technological innovation usually focus on theory research and evaluation methods. In the early stage, the evaluation system of enterprise's technological innovation was mostly constructed by questionnaire or expert interview. The influencing factors of an enterprise's technological innovation capability were obtained by the questionnaire design or expert experience. However, these methods are often affected by the limitation of the sample size and the subjective factors of expert opinions. It is difficult to objectively and comprehensively reflect the status of enterprise's technological innovation capabilities. Many scholars make a comprehensive evaluation of enterprise technological innovation. They usually use various evaluation methods to express the overall characteristics of enterprise technological innovation. These evaluation methods are mainly divided into the following aspects.

Some scholars adopted the fuzzy evaluation method to evaluate the enterprise technological innovation ability. The main feature of this method is first to design a set of evaluation index systems, determine the weight of each index, establish a fuzzy comment set, and then use the fuzzy evaluation method to judge the innovation ability of the enterprise. For example, Du et al. [15] established a risk evaluation model for technological innovation based on fuzzy evaluation. Suder and Kahraman [16] proposed a Fuzzy TOPSIS method to evaluate technological innovation investments using eight different criteria. Feng and Ma [17] identified the influencing factors of service innovation in manufacturing enterprises by using the fuzzy DEMATEL method. The shortcomings of this type of method are that human factors play a prominent role, and the data collection and processing are human-oriented, which lack objectivity and needs a lot of labour.

Some scholars used the Analytic Hierarchy Process (AHP) and its variants to evaluate the enterprise technological innovation capability. Mu et al. [18] established an index system for the technological innovation capabilities of small and medium-sized enterprises through the AHP method. Pan et al. [19] combined the AHP and osculating value process (OVP) to evaluate the green innovation ability of manufacturing enterprises. In addition, some scholars have used the improved AHP and fuzzy evaluation method to establish a model for assessing the technological innovation capabilities of enterprises [20]. The weight of each indicator in the AHP method depends on the subjective judgment of experts, and it is inevitable to have a certain degree of subjectivity.

Some researchers evaluated the enterprise technological innovation capability with the Data Envelopment Analysis (DEA) method. Wang et al. [21] constructed a high-tech industrial evaluation framework of technical innovation efficiency based on two-stage network data envelopment analysis (DEA). Ma et al. [22] used the DEA method to evaluate and analyze the innovation capability of 233 listed companies in 5 major industrial sectors defined by the China Securities Regulatory Commission (CSRC). Li et al. [23] measured the technical efficiency, scale efficiency, and pure technical efficiency of innovation in China's semiconductor industry using a three-stage DEA model. Although the DEA method can realize the multiple inputs and output, it is only an efficiency evaluation method and cannot indicate the actual technical level of the research object.

Some scholars proposed to use intelligent decision-making methods to evaluate the technological innovation capabilities of enterprises. The intelligent decision-making method applied the artificial intelligence-related theoretical methods and fusing traditional decision-making mathematical models for intelligent reasoning and solving, such as genetic algorithm, ant colony algorithm, rough set, and so on. At present, there is little literature on the application of intelligent decision-making methods to evaluate enterprise technological innovation capabilities. Shang [24] proposed the evaluation model of strategic management capability based on the Back-Propagation (BP) neural network algorithm. Zhen and Yao [25] analyzed the lean production and technological innovation in the manufacturing industry based on Support Vector Machine (SVM) algorithms and data mining technology.

Most of the related works about enterprise's technological innovation evaluation methods rely on the experts' subjective experience. Hence, the evaluation results are varied due to the different experts' opinions. Less-comprehensive impact indicators selected by experts may not reflect the actual status of the enterprise's technological innovation capability. The lack of objectivity in the evaluation method will not be conducive for identifying and cultivating enterprise's technological innovation capabilities. At present, it can objectively reflect the actual level of enterprise's technological innovation capabilities by applying big data-driven intelligent analysis methods. In addition, it can use massive and multidimensional data to establish a more comprehensive evaluation system.

## 3. Methodology

**Figure 1:**
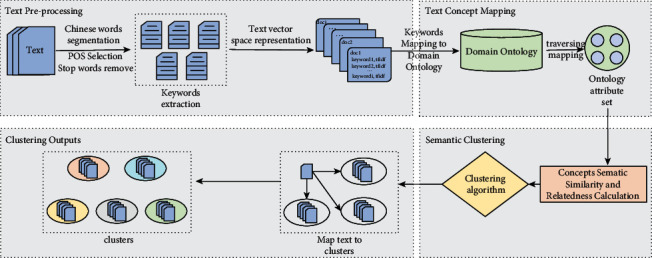
Implementation process of text clustering method based on semantic similarity and relatedness.

This paper proposed an improved semantic clustering algorithm combined with the concept of semantic similarity and relatedness based on domain ontology. After text preprocessing, a corpus containing the keywords was built and the keywords were mapped to the concepts in the domain ontology of the enterprise's technological innovation. The calculation of semantic similarity and relatedness between concepts was one of the critical steps. It needs to use the semantic similarity and relatedness method proposed in this paper to establish the compound similarity matrix. Finally, the keyword set in the corpus is clustered according to the improved semantic text clustering algorithm proposed in this paper.  Figure 1 shows the framework of semantic-based text clustering in the field of enterprise technology innovation. According to the framework, there are mainly three steps in the improved methodology and the details are as follows, and the main parameters used in the following equations are shown in Table 1.

### 3.1. Text Concept Mapping

After collecting the data from the enterprise's technological innovation, preprocessing the data, including Chinese word segmentation, custom dictionary, POS selection, and stop words removing, etc., obtained the text keyword sets. The keyword set is mapped to the ontology of the enterprise technology innovation domain to obtain the concept set. Two situations need to be considered.

1. When the dataset's keywords can directly match with the concepts in the domain ontology, the keywords *T*={*t*_1_, *t*_2_,…, *t*_*n*_} are directly mapped with the concepts *C*={*c*_1_, *c*_2_,…, *c*_*m*_}.
2. When the keywords in the dataset cannot directly match the concepts in the domain ontology and appear frequently, the keywords should be reserved as unregistered words. Calculate the occurrence frequency TF of the keyword. If TF > *μ*, keep the keyword in the unregistered word sets *W*={*w*_1_, *w*_2_,…, *w*_*l*_}, otherwise delete the keyword.

### 3.2. Semantic Similarity and Relatedness Calculation

**Figure 2:**
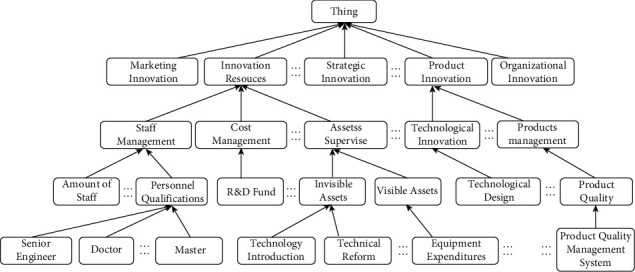
Structure of partial enterprise's technological innovation domain ontology.

Before text clustering, the semantic similarity and relatedness calculation method need to be used to construct the semantic matrix. We proposed a new semantic measurement method that combines the concepts of semantic similarity and relatedness. Firstly, calculate the semantic similarity of concepts based on the semantic distance in the established domain ontology of enterprise's technological innovation, as shown in Figure 2. Secondly, assign the weights to the path of two concepts connected in the domain ontology. The semantic distance between two concepts was obtained by traversing the sum of the weights of the connection paths instead of calculating the number of edges connecting the two concepts. The specific calculation is shown in equation (1). Then, calculate the semantic relatedness of concepts through the co-occurrence in the text. Finally, combine the semantic similarity and the relatedness to establish the compound similarity matrix *M*.

(1) $$\begin{array}{c} M=\left[\begin{array}{ccccccc} 1 & ... & \text{sim}\_{\text{rel}}_{i1} & ... & \text{sim}\_{\text{rel}}_{j1} & ... & \text{sim}\_{\text{rel}}_{l1}\\ \vdots & \ddots & ... & \vdots & ... & \vdots & \vdots \\ \text{sim}\_{\text{rel}}_{i1} & \cdots & 1 & ... & \text{sim}\_{\text{rel}}_{ji} & ... & \text{sim}\_{\text{rel}}_{li}\\ \vdots & \vdots & ... & \ddots & ... & \vdots & \vdots \\ \text{sim}\_{\text{rel}}_{j1} & ... & \text{sim}\_{\text{rel}}_{ji} & ... & 1 & ... & \text{sim}\_{\text{rel}}_{lj}\\ \vdots & \vdots & ... & \vdots & ... & \ddots & \vdots \\ \text{sim}\_{\text{rel}}_{l1} & ... & \text{sim}\_{\text{rel}}_{i1} & ... & \text{sim}\_{\text{rel}}_{lj} & ... & 1 \end{array}\right],\\ \text{Sim}\_\text{Rel}\left(c_{i},c_{j}\right)=\alpha \times \text{sim}\left(c_{i},c_{j}\right)+\left(1-\alpha \right)\times \text{rel}\left(c_{i},c_{j}\right)\\ =\alpha \times \frac{1}{1+\lambda \text{ dist}\left(c_{i},c_{j}\right)}+\left(1-\alpha \right)\times \;\log\;2\frac{f^{k}\left(c_{i},c_{j}\right)}{f^{c}\left(c_{i}\right)\times f^{c}\left(c_{j}\right)}, \end{array}$$

where *α* denotes the adjusting parameter, *λ* denotes the factor of influence degree of semantic distance on semantic similarity, dist(*c*_*i*_, *c*_*j*_) denotes the semantic distance between *c*_*i*_ and *c*_*j*_ in the domain ontology, *f*^*k*^ denotes the co-occurrence frequency of concept *c*_*i*_ and concept *c*_*j*_ in the *k* words window of the entire corpus. *f*^*c*^(*c*_*i*_) and *f*^*c*^(*c*_*j*_) represent the frequency of concept *c*_*i*_ and concept *c*_*j*_ in the whole corpus.

### 3.3. Improved K-Means Algorithm

The traditional clustering algorithm based on vector space defines the text document set as *D*={*d*_1_, *d*_2_, *d*_3_,…, *d*_*m*_} and each document can be represented as *d*_*i*_=(*w*_*i*1_, *w*_*i*2_, *w*_*i*3_,…, *w*_*in*_). The term set extracted from the document set can be expressed as *T*={*t*_1_, *t*_2_, *t*_3_,…, *t*_*n*_}. *w*_*ij*_ means the weight of the term *t*_*j*_ in the document *d*_*i*_. The traditional clustering algorithms are usually determined by the number of occurrences of the term *t*_*j*_ appearing in the document *d*_*i*_, that is, the term frequency, or the term frequency-inverse document frequency (TF-IDF), is used to assign weights. The traditional K-means algorithm selected *K* points randomly from the sample as the initial cluster centre candidate. Then, it usually calculated the distance from the sample points to the centre with the Euclidean distance formula (shown in equation (2)) and divided the points to the nearest centre. Finally, it iteratively calculated the centre of the cluster until the centre of each group does not change.

(2) $$\begin{array}{c} {\text{dis}}_{EU}\left(d_{1},d_{2}\right)=\sqrt{\left(d_{1}-d_{2}\right){\left(d_{1}-d_{2}\right)}^{T}}\\ =\sqrt{\sum_{j=1}^{n}{\left(w_{1\text{j}}-w_{2\text{j}}\right)}^{2}}. \end{array}$$

This paper proposed an improved K-means algorithm, which improves the algorithm mainly by selecting initial cluster centres and semantic-based clustering. According to the semi-positive semantic similarity and relatedness of *n* × *n* matrix *M* obtained by Step3, *M*=*W*^*T*^*W* can be obtained by orthogonalization of the positive semi-positive matrix, where the column in *W* represents the document vector. According to the semantic similarity and relatedness matrix *M*, the Euclidean distance formula can be improved as the following equation.

(3) $$\begin{array}{c} {\text{dis}}_{EU\text{ improved}}\left(d_{1},d_{2}\right)=\sqrt{\left(d_{1}-d_{2}\right){\left(Md_{1}-d_{2}\right)}^{T}}\\ =\sqrt{\left(d_{1}-d_{2}\right)W^{T}W{\left(d_{1}-d_{2}\right)}^{T}}\\ =\sqrt{\left(\left(d_{1}-d_{2}\right)W\right){\left(\left(d_{1}-d_{2}\right)W\right)}^{T}}\\ =\sqrt{\left({\hat{d}}_{1}-{\hat{d}}_{2}\right){\left({\hat{d}}_{1}-{\hat{d}}_{2}\right)}^{T}}. \end{array}$$

where ${\hat{d}}_{1}=d_{1}W$, ${\hat{d}}_{2}=d_{2}W$, the modified distance measurement considered the semantics between words based on the semantic similarity and relatedness matrix. Therefore, the distance between the document and the cluster can be measured by the definition of the following equation:

(4) $$\begin{array}{c} {\text{dis}}_{EU\text{ improved}}\left(t_{j},z_{l}\right)=\sqrt{\left(t_{j}W-z_{l}W\right){\left(t_{j}W-z_{l}W\right)}^{T}}\\ =\sqrt{\left({\hat{t}}_{j}-{\hat{z}}_{l}\right){\left({\hat{t}}_{j}-{\hat{z}}_{l}\right)}^{T}}, \end{array}$$

where *t*_*j*_ represents the feature vector of the document, *z*_*l*_ defines the cluster centre, ${\hat{t}}_{j}$ is the new document feature vector derived from *t*_*j*_, and ${\hat{z}}_{l}$ is the cluster centre derived from *z*_*l*_.

Firstly, randomly select a data point *z*_*l*_ as the initial cluster centre from the input dataset. Secondly, for each point *c*_*i*_ in the dataset, the distance from *c*_*i*_ to *z*_*l*_ is calculated by the Equation *D*(*c*_*i*_)=arg min‖*c*_*i*_ − *z*_*j*_‖_2_^2^*j*=1,2,…*k*. The point *c*_*i*_ with the maximum distance *D*(*c*_*i*_) from *z*_*l*_ is selected as the new cluster centre *z*_2_. Then, repeat the above steps until *K* initial cluster centres are found. Finally, the remaining data points in the sample set are allocated to the nearest or most similar clusters according to the principle of maximum similarity. The cluster centres in the *K* clusters are recalculated and iterated until the termination condition is met. The step of the improved K-means algorithm is described in Algorithm 1.

The improvement to the traditional K-means algorithm proposed in this paper based on semantic similarity and relevance mainly includes (1) using the improved method based on the maximum similarity to determine the initial cluster centre and reduce the random position of the cluster centre. (2) The improved Euclidean distance with semantic similarity and relatedness is used to measure the similarity between cluster centres and sample sets, instead of the traditional K-means algorithm, which ignores the semantic relationship between terms. (3) By adding convergence conditions, the original K-means algorithm solved the problem of unstable clustering results.

### 3.4. Validation

#### 3.4.1. Validation Methods

The paper mainly used the SSE and SC methods to compare the clustering results of the traditional K-means method and the improved K-means method. The SSE method calculates the total error value between any data point and the cluster centroid, and the calculation method is shown in equation (5). dis() represents the distance function, *p* is any data point in the cluster of *c*_*i*_, and *m*_*i*_ is the cluster centroid. The lower value of SSE equates to better performance of clustering. Otherwise, a higher value represents a worse clustering effect. The Silhouette Coefficient method combines clustering cohesion and separation to evaluate the effect of clustering, and the value is between [−1, 1]. The higher value of the Silhouette Coefficient indicates the better clustering performance. The calculation method of Silhouette Coefficient was shown in equation (6). *a*(*i*) represents the average distance of the data point *i* to all other points in the cluster to which the data point *i* belongs. *b*(*i*) represents the minimum value of the average distance from the data point *i* to all points of each of the other groups.

(5) $$\begin{array}{c} \text{SSE}=\sum_{i=1}^{k}\sum_{p\in c_{i}}\text{dis}{\left(p,m_{i}\right)}^{2}, \end{array}$$

(6) $$\begin{array}{c} S\left(i\right)=\frac{b\left(i\right)-a\left(i\right)}{\max\left\{a\left(i\right),b\left(i\right)\right\}}. \end{array}$$

#### 3.4.2. Validity Comparison of the Proposed Method

To compare the clustering performance between the traditional K-means algorithm based on Bag-of-words and the improved K-means algorithm based on semantic similarity and relatedness, the number of *K* is selected from 3 to 10. The experimental results of the SSE and Silhouette Coefficient are shown as follows.

**Figure 3:**
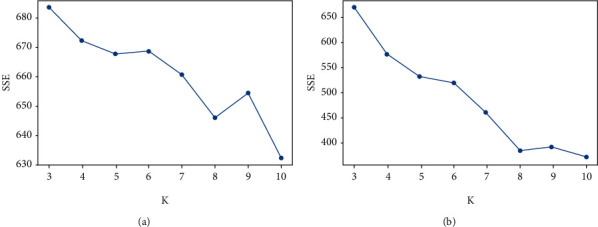
Statistical comparison of SSE. (a) Traditional K-means algorithm. (b) Semantic K-means algorithm.

Table 2 shows that as the number of *K* clusters increases, the SSE value of the improved K-means algorithm is significantly smaller than that of the traditional K-means algorithm. It shows that the clustering performance of the improved K-means algorithm is better than the traditional K-means algorithm. As shown in Figure 3, when the number of clusters (*K*) equals 8, the improved K-means algorithm and the traditional K-means algorithm have an elbow (inflexion point) within the SSE value. It shows that when the number of clusters is 8, performance clustering might be the best. It provides a reference for the value of *K* in K-means clustering.

**Figure 4:**
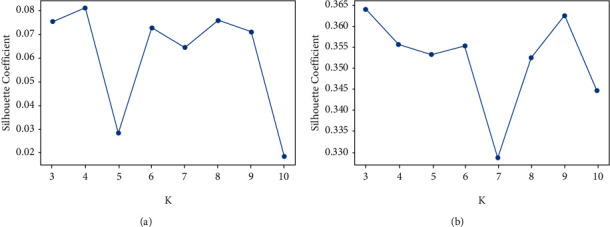
Statistical comparison of silhouette. (a) Traditional K-means algorithm. (b) Semantic K-means algorithm.

**Figure 5:**
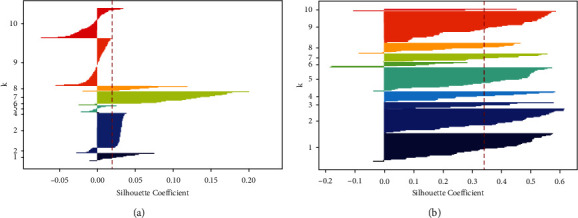
The histogram of silhouette coefficient. (a) Traditional K-means algorithm. (b) Semantic K-means algorithm.

As shown in Table 2, with the increasing number of clusters *K*, the Silhouette Coefficient value of the improved K-means algorithm is significantly higher than the traditional K-means algorithm. The higher value of the Silhouette Coefficient in the dataset indicates the better the clustering performance. Hence, it shows that the performance of the improved K-means algorithm is better than the traditional K-means algorithm. As shown in Figure 4, when the number of clusters (*K*) equals 8, the improved K-means algorithm and the traditional K-means algorithm have relatively higher values. Hence, comprehensive analysis shows that the optimum value of K is 8. The red dotted lines in Figure 5 represent the Silhouette Coefficient of the traditional K-means algorithm and improved semantic K-means algorithm. The bar chart is the category of clusters. Most of the samples in a group have a higher Silhouette Coefficient value and are distributed near the red dotted line, representing a better clustering effect. On the contrary, if the sample points have a lower Silhouette Coefficient value and the distribution is scattered, the clustering effect is worse. Figure 5 shows that the Silhouette Coefficient value of the improved K-means algorithm is higher, and the sample distributes near the red dotted line. Hence, the result indicates the performance of an improved K-means algorithm based on semantic similarity, and relatedness is better than the traditional K-means algorithm based on the Bag-of-Words model.

## 4. Experiment Results

### 4.1. Data Collection

The experimental data in this paper mainly collect the technological innovation information of 400 enterprises in Beijing and uses the document information as a text collection. The collected textual data mainly consist of the enterprise's primary status, the development of enterprise technological innovation activities, enterprise innovation projects, enterprise organizational structure, enterprise main products and services, enterprise profitability, etc.  Table 3 briefly shows the details of the data collection result.

**Figure 6:**
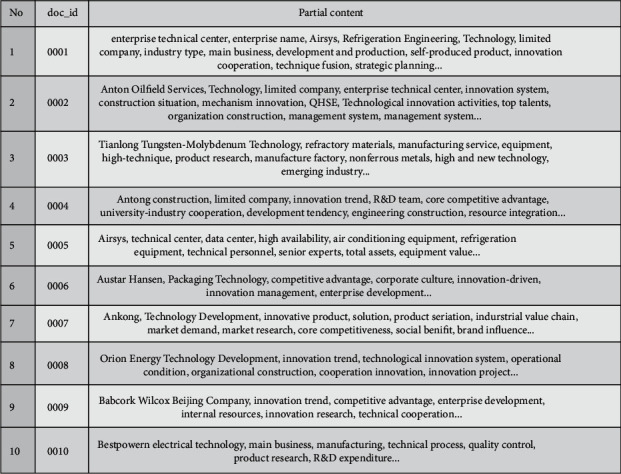
The keyword vocabulary after text preprocessing.

After data cleaning and selection, there are 867 valid texts, and the overall data size is about 20 M. The experimental operating environment is the Windows 10 system, 2.70 GHz core processor, 8.0 GB memory, and Python 3.6.2. After the preprocessing, including the custom dictionary, part-of-speech filtering, and stop words removing, the keywords vocabulary was obtained and shown in Figure 6. Then, map the keywords to the concept in domain ontology and get the semantic similarity matrix P and relatedness matrix Q by calculating the semantic similarity and relatedness.

### 4.2. Results Analysis

**Figure 7:**
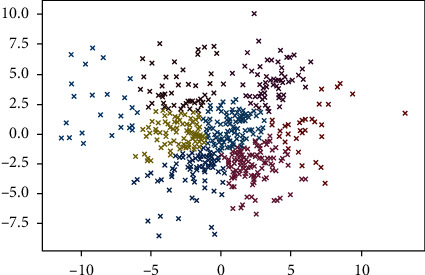
Visualization results of enterprise technology innovation text clustering when *K* = 8.

Figure 7 shows the result obtained by text clustering based on semantic similarity and relatedness. The most important 15 feature words in each cluster are selected to represent the topic based on the feature weight, as shown in Table 4. According to the topic reflected by each group, there are eight types of main factors affecting enterprise technological innovation. We combined the eighth cluster and the third cluster because they reflect the same theme. The analysis of the seven influencing factors about the technological innovation of enterprises is as follows.

  Cluster 1: manufacturing capability. The main feature words of this cluster mainly focus on the new products, new processes, new materials, process technology, equipment level, etc. The content of these feature words is related to the manufacturing capabilities of products and processes. The influence of manufacturing capability on technological innovation of enterprises is mainly reflected in the capability to transform the research and development results into manufacturing. The word “equipment level” reflects the advanced manufacturing equipment, the phrases “Construction technique, Process technology, Technical process, High-tech” reflect the topic of process design capability, and the term “quality control” reflects the content of product quality management. “Internet application, Information technology, Industrialization” reflect the theme of product innovation activities. Therefore, the cluster's words with high feature weights reflect that manufacturing capability is essential for an enterprise's technological innovation.
  Cluster 2: innovation resources. The words “engineer,” “senior engineer,” and “R&D expenditure” in this cluster have a high proportion of feature weight. The words “Engineer, Senior engineer, Senior expert, Employees number, Bachelor degree or above” reflect the quantity and quality of the R&D staff. The words “R&D expenditure, R&D, Total assets, Expenditure on science and technology activities, Main business proportion” reflect the financial investment on R&D. The word “Equipment original value” reflects the equipment investment of R&D. The phrases “Enterprise scale, Asset-liability ratio ownership structure, Technology introduction” represent the investment of enterprises in non-R&D including of enterprises own capability. The investment of innovation resources mainly refer to the quantity and quality of enterprise's investment in technological innovation resources. It is reflected in the investment of staff, funds, and equipment in R&D. The investment of innovation resources is one of the influencing factors of enterprise technological innovation.
  Cluster 3: mechanism innovation. The top feature words of this cluster can reflect directly that the topic is mainly about the mechanism innovation, which is an essential factor that affects enterprises' technological innovation. The feature words such as “Rewards system, Performance review, Post-doctor, Excellent talents, Incentive mechanism” indicate that enterprises attach importance to the incentive mechanism of personnel. The words “Organization and implementation, Operating mechanism, Organizational construction, Management system, Organizational structure” reflect the influence of the organizational management mechanism on the technological innovation of enterprises. An effective innovation mechanism can stimulate talents and cooperate effectively. The eighth group's theme is the same as cluster 3, and the sample proportion is only 4.3%. Hence, we merge the contents of cluster 3 and cluster 8.
  Cluster 4: innovation output. The feature words in this cluster, such as “Patent, Industry standard, Gross profit on sale, Main business product sales revenue,” have higher feature weight. It indicates that this cluster mainly reflects the topic of innovation output. The words “Patent, Industry standard, Science and technology progress award, Method number, Number of patent applications, Number of technology development projects, Number of new product development projects, Software copyright, Utility model, Design patent” reflect the technological output of enterprise's innovation. The words “Gross profit on sale, Main business product sales revenue, Industrial output, Industrial added value” reflect the innovation benefits. Therefore, from the distribution of feature words, the innovation output is also an important performance that affects the technological innovation of an enterprise.
  Cluster 5: market innovation. The words “Competitive advantage, Social benefit, Market competitiveness” have high feature value in this cluster. The keywords tend to reflect market innovation, which means product sales and promotion innovations can meet market demands. With the continuous expansion of market scale and the increase of market demand, the market-oriented product sales innovation model has become one of the important factors affecting the development of enterprise technological innovation.
  Cluster 6: protection measures. “Intellectual property, Intellectual property protection, Intellectual property management, Independent intellectual property” are high feature value words in this cluster, reflecting the content of intellectual property protection measures. Technical knowledge protection can promote technology diffusion and ensure attracting foreign capital and technology introduction. Therefore, the protection measures for intellectual property and technology are conducive to promoting the technological innovation of enterprises and are an important influencing factor.
  Cluster 7: innovation strategy. This cluster contains the most significant proportion of samples. The keywords “Industry-University-Research Cooperation, Industry-University-Research, Internal and external resources, Integration, Resource Integration” reflect the topic of innovation strategy. It refers to the integration and arrangement of internal and external innovation resources and technologies based on the enterprise's overall strategy with enterprise operation. The phrases “Industry-University-Research Cooperation, Industry-University-Research, Research institutes, Colleges and universities, R&D team” represent the content of the joint innovation strategy of enterprises and industry, university, and research. The phrases “Internal and external resources, Resource Integration” reflect the integration and allocation of internal and external resources. The terms “Technical cooperation, Technology Exchange, Technology fusion” reflect the strategic plan of enterprises for technology integration and innovation. The phrases “Core competence, Strategic planning, Overall planning, Leader” represent the innovation capability and strategy of the enterprise leader or decision-maker. Therefore, the topic of this cluster is innovation strategy, and the correctness of the innovation strategy also has an important impact on enterprise technological innovation.

**Figure 8:**
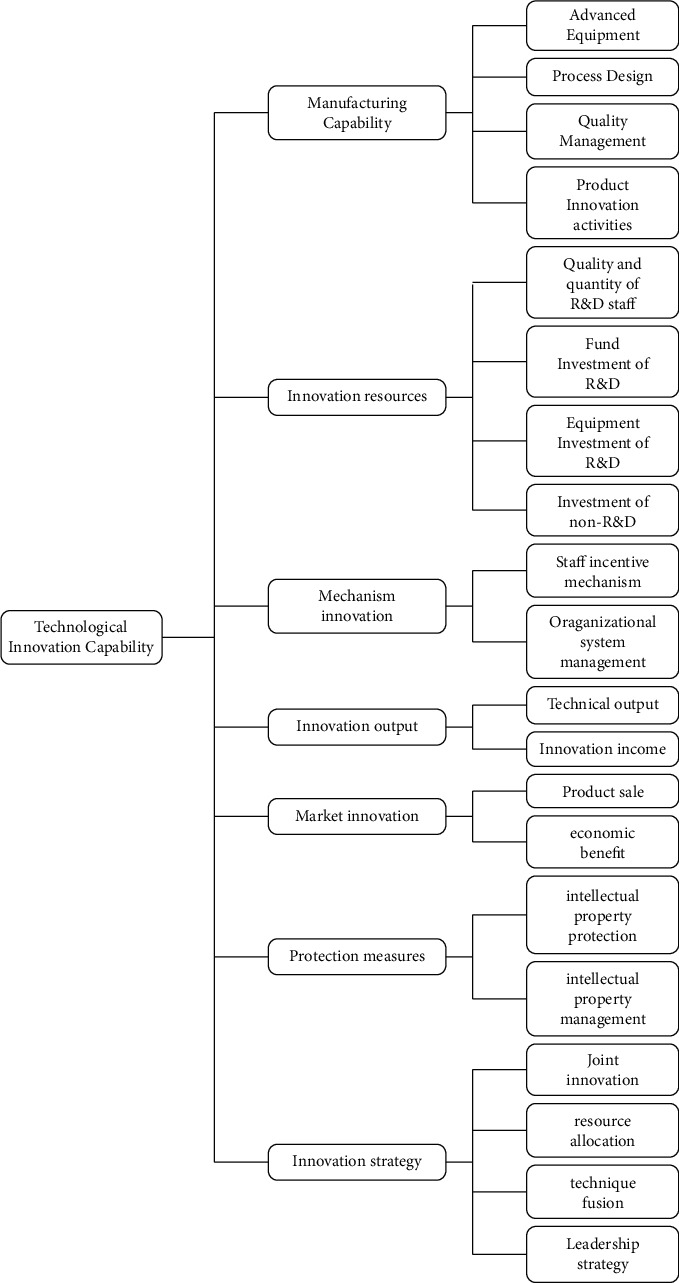
The framework of influencing factors of enterprise's technological innovation.

Figure 8 shows the framework of the influencing factors for enterprises' technological innovation. There are 7 types of impacts factors: manufacturing capability, innovation resource, mechanism innovation, innovation output, market innovation, protection measures, and innovation strategy. Through analysis of the linking feature words of each cluster, the meso-level concept can be concluded, as shown in Figure 8.

## 5. Discussion

### 5.1. Policy Suggestions

It can be seen from the framework of influencing factors of enterprise's technological innovation, and the four clusters results that are particularly prominent include the “Protection measure,” “Innovation strategy,” “Market innovation,” and “Mechanism innovation” except the “Manufacturing capability,” “Innovation resources,” and “Innovation output,” which are included in most of the researches. The four aspects are relatively new in the field of enterprise's technological innovation. Thus, the paper provides policy suggestions from these four aspects for the enterprise's technological innovation.

The innovation capability of intellectual property is an important aspect to measure the innovation output of enterprises. In the current fierce competition environment, enterprises are rushing to develop scientific and technological achievements through various channels for survival and development, and seek legal protection by applying for patents. However, it is not uncommon for enterprises to suffer heavy losses because their competitors registered the patents and trademarks in advance. Therefore, enterprises should strengthen their awareness of intellectual property protection, set up special intellectual property departments, and pay attention to cultivating professional intellectual property talents.

The innovation strategy is a plan and methodology for enterprises to develop new products and services in the future. It aligns the development of innovations with future corporate goals, which requires formulation based on the external environment and internal conditions. Thus, the innovation strategy directly reflects leadership decision-making. The weak sense of innovation or lack of innovative decision-making power for senior decision-makers will bring risks to the enterprise. The enterprises should plan and layout the innovative strategic cooperation in advance. In addition, it strengthens the collaboration between schools and industry and establishes strategic partners, which is helpful for the enterprise's technological innovation.

Market innovation is mainly reflected in marketing and management capabilities. From the formation of initial products to the market introduction, the whole process is inseparable from professional marketers and marketing strategies. Therefore, to strengthen the market development, enterprises need to expand sales channels, explore new sales models, and improve the sales platforms. Moreover, enterprises should build their own sales team, introduce professional marketing personnel, and strengthen the training and management of marketing.

Introduce innovative management talents and improve the enterprise innovation mechanism. The enterprises need to construct a suitable organizational framework and formulate the innovation management system, including a talent introduction mechanism, talent training mechanism, innovation incentive system, reasonable innovation evaluation system, innovation achievements protection mechanism, etc. It can mobilize the enthusiasm of corporate managers and employees by improving the incentive mechanism, including interest incentives, competence incentives, power incentives, and responsibility incentives. The interest incentives include salary, welfare, bonus, etc. Competence incentives include training, competitive employment, etc. The power incentives mainly refer to promotion, and responsibility incentives mainly refer to a reasonable assessment system. Mobilizing the enthusiasm and creativity of employees can effectively enhance the enterprise's technological innovation capabilities.

### 5.2. Implication for Theory and Practice

This paper enriches and deepens the theoretical research on evaluating enterprise's technological innovation capability from the theory aspect. The paper first extracts prominent factors that affect enterprise technological innovation based on the collected textual data. It provides a reference for the construction method of the enterprise's technological innovation evaluation index system. Moreover, the process of enterprise's technological innovation is dynamic and continuous. Traditional assessment methods for enterprise technology innovation lack automatic processing ability and cannot meet the needs of large-scale, high-quality, and in-depth knowledge acquisition. As a high-tech knowledge processing technology, the text mining method has significant advantages in the intelligent analysis of enterprise technological innovation capabilities. It can reflect the objective level of enterprise's technological innovation under the data-driven. This paper proposes a method for discovering enterprise technological innovation knowledge based on semantic mining technology. It is helpful to explore the potential factors of influencing the enterprise technological innovation by revealing the inherent complex associations of enterprise's textual data and extracting valuable patterns and knowledge. To our knowledge, this research is the first attempt to apply the semantic text clustering algorithm in enterprise's technological innovation. In addition, this paper analyzed the development status and trends of the enterprise's technological innovation capabilities based on the collected textual data from 400 enterprises in Beijing. It helps to promote technological innovation of the enterprises by smart analysis under the data-driven. Thus, the potential benefits of the proposed model can help drive and facilitate the enterprise's technical innovation capability.

## 6. Conclusions

This paper utilizes a data-driven intelligent mining method based on a semantic conceptual model to analyze the influencing factors of enterprise technology innovation. Some scholars believe that technological innovation's evaluation system construction method is a questionnaire design and survey analysis. However, it inevitably involves human factors and the subjective judgments of experts. This study proposed a systematic process for evaluating the enterprise's technological innovation based on sizeable textual data. Computer information processing analyzes the evaluation factors and indicators that affect enterprise technology innovation from objective data. Furthermore, the traditional text clustering algorithm based on the Bag-of-words model ignores the semantic relationship between concepts, resulting in an unsatisfactory clustering effect. Therefore, this paper proposed an improved semantic concept-based clustering algorithm to analyze enterprise's collected textual data. The performance of the improved K-means clustering method based on semantic similarity and relatedness is superior to the traditional K-means clustering method. The proposed method realized the clustering of critical factors in the field of enterprises technological innovation, and the analysis of experimental results can obtain the seven key factors that affect the technological innovation of enterprises.

## Figures and Tables

**Algorithm 1:**
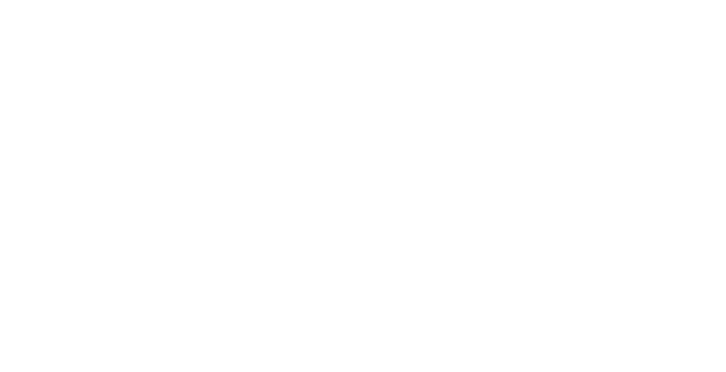
Improved semantic similarity and relatedness-based K-means clustering algorithm.

**Table 1:** The main parameters in equations' definitions.

Elements	Definition
*t*_*n*_	Text keywords
*c*_*m*_	Concepts in the domain ontology
*w*_*l*_	Unregistered word
TF	The frequency of the keyword in dataset
*μ*	The threshold value of keyword frequency
*M*	The semantic similarity and the relatedness matrix
dist(*c*_*i*_, *c*_*j*_)	The semantic distance between concepts *c*_*i*_ and *c*_*j*_ in ontology
*λ*	The influence factor of semantic distance on semantic similarity
*f* ^*k*^(*c*_*i*_, *c*_*j*_)	The number of times that concept *c*_*i*_ and *c*_*j*_ appear simultaneously in the *k* words window at the entire corpus
*f* ^*c*^(*c*_*i*_)	The frequency of the concept *c*_*i*_ at the entire corpus
rel(*c*_*i*_, *c*_*j*_)	Relatedness between concept *c*_*i*_ and *c*_*j*_
Sim_Rel(*c*_*i*_, *c*_*j*_)	Semantic similarity and relatedness between concept of *c*_*i*_ and *c*_*j*_
*α*	The adjusting parameter
*d*_*i*_	The text document
*w*_*ij*_	The weight of the term *t*_*j*_ in the document *d*_*i*_
*t*_*j*_	The feature vector of the document
*z*_*l*_	The cluster centre
${\hat{t}}_{j}$	The new document feature vector derived from *t*_*j*_
${\hat{z}}_{l}$	The cluster centre derived from *z*_*l*_

**Table 2:** The performance comparison between improved K-means algorithm based on semantic and traditional K-means algorithm.

*K* Value	SSE		Silhouette coefficient	
	Traditional K-means algorithm based on bag-of-word model	Improved K-means algorithm based on semantic similarity and relatedness	Traditional K-means algorithm based on bag-of-word model	Improved K-means algorithm based on semantic similarity and relatedness
3	683.2779	672.4002	0.075280	0.363640
4	671.9479	577.2000	0.081084	0.355436
5	667.6426	532.9228	0.028152	0.352955
6	668.5414	519.1228	0.072469	0.355115
7	660.5026	461.7781	0.064319	0.328729
8	**645.9339**	**385.5498**	**0.075842**	**0.352197**
9	654.0991	392.1088	0.071101	0.362227
10	632.2624	373.0217	0.018480	0.344523

When the value of *K* equals 8, the improved *K*-means algorithm and the traditional *K*-means algorithm have a relatively higher value.

**Table 3:** Summaries of data collection.

Area of focus	Format	Number
Enterprise profiles	.doc	388
Enterprise technical and financial reports	.xlsx	212
Enterprises products	.txt	131
Enterprises rewards	.pdf	136

**Table 4:** The text clustering result of enterprise's technological innovation when *K* = 8.

Cluster	Proportion of included samples (%)	Top 15 topic feature words
Cluster 1	16.1	New product, Internet application, new technology, construction technique, information technology, new technique, advanced level, composite material, design and development, quality control, process technology, technical process, industrialization, high-tech, equipment level, individuation
Cluster 2	16	Engineer, senior engineer, senior expert, R&D expenditure, R&D, total assets, employees' number, equipment original value, expenditure on science and technology activities, main business proportion, bachelor degree or above, enterprise scale, asset-liability ratio ownership structure, technology introduction
Cluster 3	19.2	Mechanism innovation, organization and implementation, operating mechanism, rewards system, organizational construction, management system, organizational structure, performance reviews, post doctor, rules and regulations, excellent talents, organization, incentive mechanism, communication channel, guarantee mechanism
Cluster 4	9.7	Patent, industry-standard, gross profit on sale, main business product sales revenue, science and technology progress award, method number, number of patent applications, industrial output, industrial added value, platform, number of technology development projects, number of new product development projects, software copyright, utility model, design patent
Cluster 5	7.3	Competitive advantage, social benefit, brand influence, market competitiveness, core competence, reputation, diversification, business area, marketing, market-driven, market demand, development trend, market occupancy, market share, products sale
Cluster 6	5.7	Intellectual property, intellectual property protection, intellectual property management, independent intellectual property, intellectual property application, property rights transformation, transfer of property rights, license, patent warning, confidential agreement, trade secrets, technology protection, protection method, promote application, intellectual property layout
Cluster 7	21.7	Industry-university-research cooperation, industry-university-research, internal and external resources, integration, resource integration, core competence, strategic planning, research institutes, colleges and universities, R&D team, technical cooperation, technology exchange, overall planning, leader, technology fusion
Cluster 8	4.3	Technical staff, innovation mechanism, management mechanism, director of technology centre, manager, scientists, technology leader, positivity, talent team, innovation team, outstanding contribution, postgraduate, technical experts, talent incentive mechanism, youth technology leader

## Data Availability

The data used to support the findings of this study are available from the corresponding author upon request.
